# Antimicrobial and antiproliferative activities of stingless bee *Melipona scutellaris* geopropolis

**DOI:** 10.1186/1472-6882-13-23

**Published:** 2013-01-28

**Authors:** Marcos Guilherme da Cunha, Marcelo Franchin, LíviaCâmaradeCarvalho Galvão, AnaLúciaTascaGóis de Ruiz, João Ernesto de Carvalho, Masarahu Ikegaki, Severino Matias de Alencar, Hyun Koo, Pedro Luiz Rosalen

**Affiliations:** 1 Department of Physiological Sciences, Piracicaba Dental School, University of Campinas UNICAMP, Av. Limeira 901, 13414-903, Piracicaba, SP, Brazil; 2 Research Center for Chemistry, Biology, and Agriculture, University of Campinas –UNICAMP, CP 6171, 13083-970, Campinas, SP, Brazil; 3 College of Pharmaceutical Sciences, Federal University of Alfenas, 37130–000, Alfenas, MG, Brazil; 4 Department of Agri-Food Industry, Food, and Nutrition, “Luiz de Queiroz” College of Agriculture, University of São Paulo, Av. Pádua Dias, 11, Piracicaba, SP, Brazil; 5 Center for Oral Biology, University of Rochester, 601 Elmwood Avenue, Rochester, NY, 14642, USA

**Keywords:** Melipona scutellaris, Geopropolis, Antimicrobial activity, Antiproliferative activity, Chemical profile

## Abstract

**Background:**

Geopropolis is a type of propolis containing resin, wax, and soil, collected by threatened stingless bee species native to tropical countries and used in folk medicine. However, studies concerning the biological activity and chemical composition of geopropolis are scarce. In this study, we evaluated the antimicrobial and antiproliferative activity of the ethanolic extract of geopropolis (EEGP) collected by *Melipona scutellaris* and its bioactive fraction against important clinical microorganisms as well as their *in vitro* cytotoxicity and chemical profile.

**Methods:**

The antimicrobial activity of EEGP and fractions was examined by determining their minimum inhibitory concentration (MIC) and minimum bactericidal concentration (MBC) against six bacteria strains as well as their ability to inhibit *Streptococcus mutans* biofilm adherence. Total growth inhibition (TGI) was chosen to assay the antiproliferative activity of EEGP and its bioactive fraction against normal and cancer cell lines. The chemical composition of *M. scutellaris* geopropolis was identified by reversed-phase high-performance liquid chromatography and gas chromatography–mass spectrometry.

**Results:**

EEGP significantly inhibited the growth of *Staphylococcus aureus* strains and *S. mutans* at low concentrations, and its hexane fraction (HF) presented the highest antibacterial activity. Also, both EEGP and HF inhibited *S. mutans* biofilm adherence (p < 0.05) and showed selectivity against human cancer cell lines, although only HF demonstrated selectivity at low concentrations. The chemical analyses performed suggest the absence of flavonoids and the presence of benzophenones as geopropolis major compounds.

**Conclusions:**

The empirical use of this unique type of geopropolis by folk medicine practitioners was confirmed in the present study, since it showed antimicrobial and antiproliferative potential against the cancer cell lines studied. It is possible that the major compounds found in this type of geopropolis are responsible for its properties.

## Background

Propolis, a resin collected by bees from several plants, presents a great variety of pharmacological effects already described in the literature, such as antimicrobial, anti-inflammatory, immune-modulatory, anti-ulcer, and anti-tumor properties. Regarding antimicrobial activity, several types of propolis collected by *Apis mellifera* seem to be active against various microorganisms [1]. The variation in biological activity of different types of propolis is directly related to their complex chemical composition, which can vary according to season, region of plant resin collection [2], and bee species. Most of the studies available in the international literature, however, are related to propolis collected by *A. mellifera*, whereas other types of propolis collected by different species of bees have been sparsely studied.

Geopropolis is a different kind of propolis because it presents wax and soil in its constitution, conferring unique characteristics to it. This type of propolis, collected by stingless bees, such as *Melipona scutellaris*, an endangered bee species native to tropical countries, has been scarcely described in the literature and little is known about its chemical composition and biological activity. Despite being widely used by low income communities in Brazil, especially in the Northeast Region, this substance is not a value added product from beekeeping [3].

Velikova et al. [4] described the antimicrobial activity of samples of Brazilian geopropolis against *Staphylococcus aureus* and *Escherichia coli*, suggesting the presence of nonpolar compounds that would account for this property. Liberio et al. [5] showed that geopropolis from Maranhão, Brazil, collected by *Melipona fasciculate*, presented antimicrobial activity against *S. aureus* and *Candida albicans*, and also exhibited bactericidal effects against *Streptococcus mutans* biofilms. Nonetheless, the antimicrobial activity was attributed only to samples with the highest flavonoid contents. Given that the geopropolis collected by this bee species exhibits interesting antimicrobial profile, elucidating its biological and chemical characteristics is of fundamental importance to characterize the potential use of this not fully studied type of propolis as medicine or functional food.

Bacteria that normally inhabit the oral cavity, such as *S. mutans*, *Actinomyces naeslundii,* and *Enterococcus faecalis*, acquire relevant clinical importance in opportunistic pathogenic situations, since they may be related to several oral infections. Among these microorganisms, *S. mutans* deserves special attention due to its unique ability to form biofilms, and consequently start the cariogenic process [6] or endocarditis [7]. Also, bacteria such as *S. aureus,* methicillin-resistant *S. aureus* (MRSA), and *Pseudomonas aeruginosa* are often associated with nosocomial infections and have been showing increased resistance to many available antibiotics [8] stimulating new approaches for alternative treatments.

Once a natural product is proven to present antimicrobial activity, it is necessary to know whether it has compatibility with the normal cells of the host to enable its possible harmless use. Furthermore, some authors have studied natural products which act against microorganisms and also exhibit antiproliferative activity against tumor cells, increasing the range of possible uses for these products [9]. A type of propolis found in the tropical region and collected by stingless bee species showed antiproliferative activity *in vitro* against tumor but not normal cell lines [10].

Given the lack of scientific information about geopropolis collected by *M. scutellaris*, this study aimed to evaluate the antimicrobial and antiproliferative activity of the ethanolic extract of geopropolis (EEGP) and its fractions, as well as characterize them chemically, thereby generating reliable information that may add value to this natural product.

## Methods

### Geopropolis sample and fractionation

Crude samples of *M. scutellaris* geopropolis were obtained in the city of Entre Rios, in the state of Bahia (11°57' S, 38°05' W), Northeast Region of Brazil. The geopropolis sample (100 g) was extracted with absolute ethanol (1:7, w/v), at 70°C, for 30 min and then filtered to obtain its ethanolic extract (EEGP). The EEGP was fractionated by liquid–liquid extraction, based on a polarity gradient, and hexane (HF), chloroform (CF), and ethyl acetate (EAF) fractions were obtained, as previously detailed by Franchin et al. [11]. The fractions obtained were monitored by thin layer chromatography (TLC) using the anisaldehyde reagent, followed by incubation at 100°C for 5 min. The fluorescent substances were visualized under ultraviolet (UV) light at the wavelengths of 254 nm and 366 nm. EEGP, HF, CF, and EAF were concentrated and yields of 4.33 (w/w), 1.98 (w/w), 0.23 (w/w), and 0.87 (w/w) were obtained, respectively. EEGP and all the fractions were reconstituted with absolute ethanol to 3.2% (w/v) before further use. Since geopropolis presents soil in its composition and this can contain antimicrobial substances [12], samples of the soil around the hive underwent the same process of extraction with absolute ethanol used to obtain EEGP, and had their antimicrobial activity evaluated.

### Bacterial strains and susceptibility testing

The bacterial strains used in this study were: *Streptococcus mutans* UA 159, *Staphylococcus aureus* ATCC 25923, *Staphylococcus aureus* ATCC 33592 (methicillin-resistant *Staphylococcus aureus*), *Enterococcus faecalis* ATCC 29212, *Actinomyces naeslundii* m104, and *Pseudomonas aeruginosa* ATCC 25619. The antimicrobial activity of EEGP and fractions was examined by determining the minimum inhibitory concentration (MIC) and minimum bactericidal concentration (MBC), in accordance with the Clinical and Laboratory Standards Institute (CLSI) guidelines [13]. MIC was performed in 96-well microplates, inoculated with 5 × 10^5^ CFU/mL, using brain heart infusion medium (BHI, Difco, Franklin Lakes, NJ, USA), and the concentrations of EEGP and fractions ranged from 3.125 to 1600 μg/mL. The vehicle control was ethanol (final ethanol concentration: 5%, v/v), and the positive control was 0.12% chlorhexidine digluconate (Sigma-Aldrich, St. Louis, MO, USA). The plates were incubated at 37°C and 5% CO_2_ for 24 h and MIC was defined as the lowest concentration of EEGP or fraction that allowed no visible growth, confirmed by 0.01% resazurin dye (Sigma-Aldrich, St. Louis, MO, USA). MBC was determined by sub-culturing on BHI agar 50-μL aliquots of each incubated well that presented concentration higher than the MIC [14]. Three separate experiments were conducted in triplicates for each concentration of EEGP and fractions.

### Inhibition of *Streptococcus mutans* biofilm adherence

The ability of EEGP and its bioactive fraction (HF) to inhibit the adherence of *S. mutans* growing cells was tested as described by Castro et al. [15] and Galvão et al. [16] with some modifications. Briefly, *S. mutans* cells (1.0 × 10^5^ CFU/mL in BHI plus 1% sucrose w/v) were grown in 96-well sterilized polystyrene U-bottom microtiter plates containing EEGP or HF at sub-MIC concentrations or the vehicle control (5% v/v ethanol). After incubation at 37°C for 18 h, the adherent cells were stained with 1% crystal violet aqueous solution (w/v) and resuspended in absolute ethanol. Biofilm formation was quantified by measuring the absorbance at 575 nm using a Biochrom Asys UVM 340 Scanning Microplate Reader (Asys HiTech GmbH, Cambridge, United Kingdom) and the ScanPlus 2.0.1 software.

### Antiproliferative assay

The *in vitro* antiproliferative assay was performed according to Monks et al. [17]. Also, The human keratinocyte cell line HaCaT, kindly donated by Dr. Ricardo Della Coletta (FOP, UNICAMP, Piracicaba, SP, Brazil), murine normal fibroblast (3T3) and eight human tumor cell lines [glioma (U251), melanoma (UACC-62), breast (MCF-7), multidrug resistant ovarian (NCI-ADR/RES), kidney (786–0), lung, non-small cells (NCI-H460), prostate (PC-3), and ovarian (OVCAR-03)], kindly provided by the National Cancer Institute (Frederick, MD, USA), were used in this study. Stock and experimental cultures were grown in medium containing 5 mL RPMI 1640 (Gibco BRL, Gaithersburg, MD, USA) supplemented with 5% fetal bovine serum (Gibco BRL). Peniciline:streptomicine mixture (1000 U/mL:1000 μg/mL, 1 mL/L RPMI) was added to the experimental cultures. In 96-well plates, 100 μL cells/well of each cell line aforementioned were exposed to EEGP and HF at the concentrations of 0.25, 2.5, 25, and 250 μg/mL in dimethyl sulfoxide (DMSO)/RPMI, vehicle control, or doxorubicin (Dox) used as positive control (0.25, 2.5, 25, and 250 μg/mL), at 37°C, 5% CO_2_ aerobically for 48 h. Final DMSO concentration did not affect cell viability. Before (T0 plate) and after sample addition (T1 plates), cells were fixed with 50% trichloroacetic acid and cell proliferation was determined by spectrophotometric quantification (540 nm) of cellular protein content using the sulforhodamine B assay. Three measurements were obtained at the beginning of incubation (time zero, T0) and 48 h postincubation for compound-free (C) and tested (T) cells. Cell proliferation was determined according to the equation 100 × [(T-T0)/C-T0], for T0 < T ≤ C, and 100 × [(T-T0)/T0], for T ≤ T0. A concentration-response curve was plotted for each cell line and, from these curves, TGI (concentration that promotes total growth inhibition) was determined by the concentration-response curve for each cell line obtained by non-linear regression analysis using the software Origin 8.0 (OriginLab Corporation, Inc., Northhampton, MA, USA).

### Chemical assays

#### Reversed-phase high-performance liquid chromatography (RP-HPLC)

EEGP was analyzed by reversed-phase high-performance liquid chromatography (RP-HPLC) using a liquid chromatography system (Shimadzu Ltd., Kyoto, Japan) with a Shimadzu ODS-A column (RP-18, 4.6 mm × 250 mm; 5 μm particle size) and a photodiode array detector (SPD-M10AVp) at 254 nm. EEGP was filtered through 0.22 mm filter (Millipore) and 20 mL injected into the HPLC system. The column was eluted by using a linear gradient of water/acetic acid (19:1, v/v) (solvent A) and methanol (solvent B) at a constant flow rate of 1 mL/min. The gradient started with 30% solvent B, changing to 40% of B in 15 min, 50% of B in 30 min, 60% of B in 45 min, 75% of B in 65 min, 75% of B in 85 min, 90% of B in 95 min, 90% of B in 110 min, and 30% of B in 120 min. The column was maintained at 35°C. The chemical compounds were identified by their absorption spectra in the UV region, using the photodiode array detector and comparison to authentic standards (p-coumaric acid, ferulic acid, cinnamic acid, gallic acid, quercetin, kaempferol, kaempferide, apigenin, sakuranetin, isosakuranetin, pinocembrin, chrysin, acacetin, and galangin).

#### Derivatization – formation of trimethylsilyl derivatives (TMS)

Prior to gas chromatography–mass spectrometry **(**GC-MS) analysis, EEGP underwent the crucial stage of chemical derivatization, widely used to reduce the polarity of functional groups and facilitate their separation during GC-MS analysis. An aliquot of 10 mg of EEGP was added to 100 μL of the derivatizing reagent N-methyl-N-trimethylsilyltrifluoroacetamide (MSTFA) and the reaction mixture was homogenized and incubated at 70°C for 10 min. The reagent was evaporated under a stream of nitrogen and trimethylsilyl (TMS) derivatives were rediluted in hexane (800 μL). After homogenization, the supernatant was transferred to a vial and injected into the GC-MS system.

#### Gas chromatography–mass spectrometry (GC-MS)

GC-MS analysis of the derivatized EEGP was performed using gas chromatography system GC-2010 (Shimadzu Ltd., Kyoto, Japan), coupled to a mass spectrometer (QP 2010 Plus, Shimadzu Ltd., Kyoto, Japan). The temperature program started at 60°C (1 min), increasing at 3°C/min to 240°C, remaining at 240°C for 15 min. Helium was used as the carrier gas, the injector temperature was 280°C, and the injection volume was 0.5 μL in splitless mode. The interface was maintained at 280°C and the detector was operated in the scanning mode (m/z 40–800). Data integration was performed using the LabSolutions-GCMS software. Flavonoids, phenolic acids, and derivatives were identified by comparing their retention time and ion fragmentation with coded and authentic standards (quercetin, apigenin, kaempferol, kaempferide, rutin, epicatechin, catechin, resveratrol, ferulic acid, caffeic acid, p-coumaric acid, and cinnamic acid) eluted under the same conditions as well as with the Wiley Version 8 library [18].

#### Statistical analysis

The results obtained for inhibition of *S. mutans* biofilm adherence were compared using Kruskal-Wallis test (p < 0.05 was considered statistically significant). Triplicates from at least three separate experiments were conducted for each assay.

## Results

Table 1 shows MIC and MBC values for EEGP and fractions against the tested microorganisms. EEGP was able to inhibit the growth of *S. mutans*, *S. aureus*, and MRSA strains at concentrations lower than 50 μg/mL, while the growth of *E. faecalis* and *A. naeslundii* was inhibited between 800 and 1600 μg/mL. Neither EEGP nor the fractions inhibited the growth of *P*. *aeruginosa* at the tested concentrations. Except for *S. aureus* strains, which were killed between 25 and 50 μg/mL, MBC values showed that EEGP presented bactericidal activity at concentrations over 1600 μg/mL against the tested microorganisms. The extract of the soil obtained from the region of geopropolis collection showed the same antimicrobial profile of the vehicle, thus not interfering with the growth of the tested microorganisms.

**Table 1:** **Minimum inhibitory concentration (MIC) and minimum bactericidal concentration (MBC) values of the ethanolic extract of geopropolis (EEGP) and its fractions (hexane – HF; chloroform – CF; ethyl acetate – EAF) against the tested microorganisms (values in** μ**g/mL)**

**Microorganism**	**EEGP**		**HF**		**CF**		**EAF**	
	**MIC**	**MBC**	**MIC**	**MBC**	**MIC**	**MBC**	**MIC**	**MBC**
*Streptococcus mutans* UA 159	25–50	^a^	6.25–12.5	800–1600	25–50	^a^	^a^	^a^
*Staphylococcus aureus* ATCC 25923	6.25–12.5	25–50	6.25–12.5	25–50	12.5–25	50–100	50–100	100–200
*Staphylococcus aureus* ATCC 33592 (MRSA)	6.25–12.5	25–50	12.5–25	25–50	6.25–12.5	25–50	25–50	50–100
*Enterococcus faecalis* ATCC 29212	800–1600	^a^	100–200	800–1600	400–800	^a^	400–800	^a^
*Actinomyces naeslundii* m104	800–1600	^a^	200–400	800–1600	400–800	^a^	400–800	^a^
*Pseudomonas aeruginosa* ATCC 25619	^a^	^a^	^a^	^a^	^a^	^a^	^a^	^a^

^a^ Value > 1600 μg/mL.

The geopropolis fractions were tested to observe whether the chemical separation process was able to reduce their MIC values in relation to EEGP. Table 1 shows that, compared to EEGP, HF (nonpolar) presented lower or equal MIC values for *S. mutans*, *S. aureus,* and MRSA strains, and for *E. faecalis* and *A. naeslundii*, this value was reduced to 100–200 μg/mL and 200–400 μg/mL, respectively.

**Figure 1:**
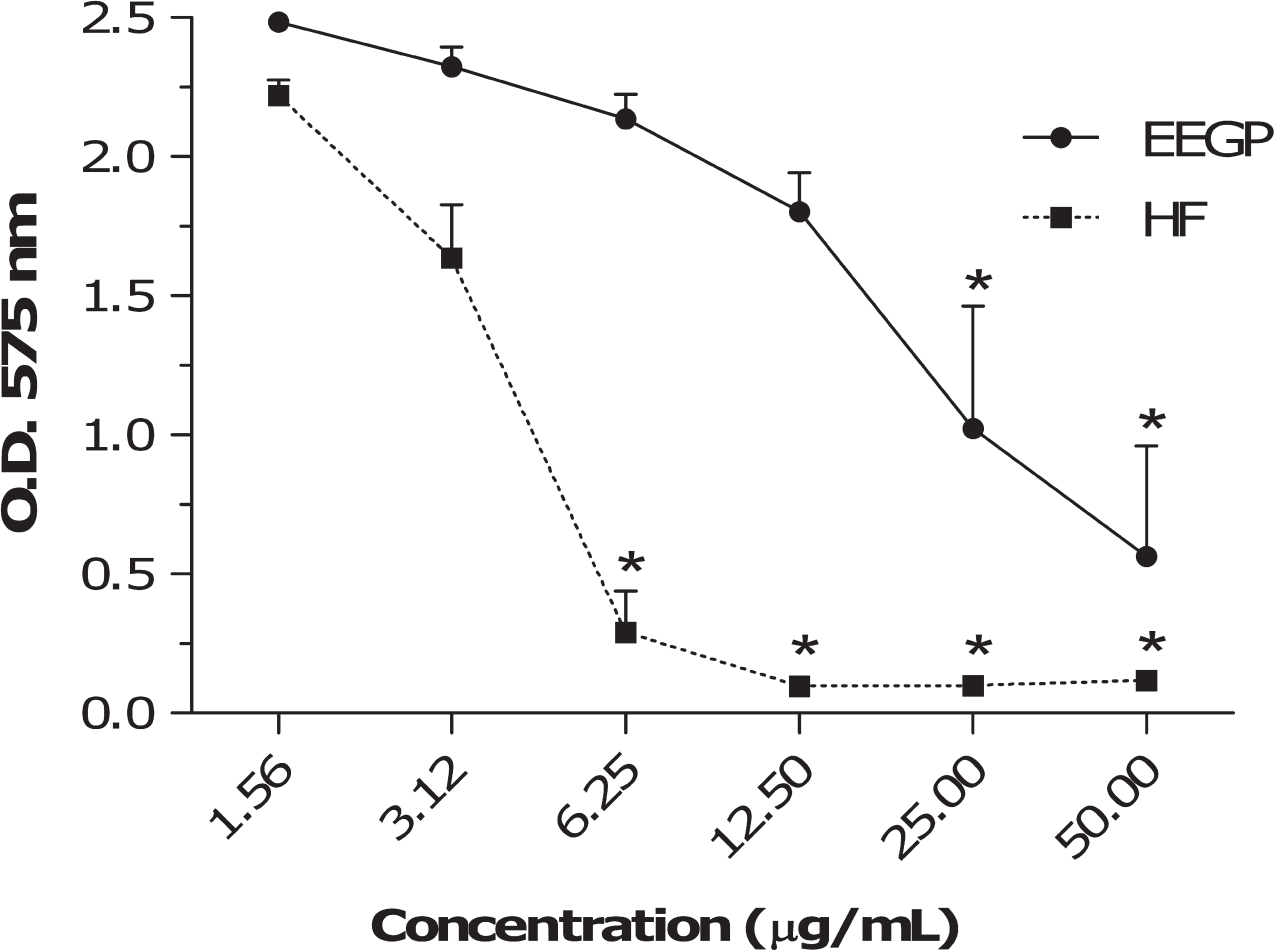
**Effect of the ethanolic extract of geopropolis (EEGP) and its hexane fraction (HF) to inhibit the adherence of *Streptococcus mutans *UA159 growing cells.** Each concentration marked with * differs significantly from the vehicle control (p < 0.05, ANOVA, Student-Newman-Keuls).

Figure 1 shows that both EEGP and HF were able to significantly decrease (p < 0.05) *S. mutans* biofilm formation. The lowest concentrations of EEGP and HF that significantly reduced the biofilm formation were 25 and 6.25 μg/mL, respectively. EEGP showed inhibition rate of 51% at 25 μg/mL, whereas HF showed inhibition rate of 86% at 6.25 μg/mL compared to the vehicle control.

Table 2 shows the antiproliferative activity of EEGP and HF against normal and tumor human cell lines. EEGP presented more activity against tumor cell lines, inhibiting the total growth at low concentrations when compared to normal lines. All tumor cell lines tested were inhibited by EEGP at concentrations below 35 μg/mL, whereas the normal cell lines (HaCaT and 3T3) were inhibited over 40 μg/mL (43.20 and 52.73 μg/mL, respectively). The lowest TGI value for EEGP was observed against melanoma tumor cells (10.90 μg/mL). The TGI values obtained for HF were lower than 15.00 μg/mL for most of the cell lines tested and 32.00 μg/mL for HaCaT. HF was also more selective regarding the melanoma line, since the TGI was 1.77 μg/mL, about six times lower than that registered for EEGP.

**Table 2:** Total growth inhibition (TGI) of the ethanolic extract of geopropolis (EEGP), its hexane fraction (HF), and the positive control doxorubicin (Dox) on human normal and tumor cell lines

**Cell line**	**TGI (**μ**g/mL)**		
	**EEGP**	**HF**	**Dox**
Keratinocytes (HaCaT)^a^	43.20	32.00	0.96
Murine normal fibroblast (3T3)^a^	52.73	12.27	0.92
Glioma (U251)	21.18	7.17	1.08
Melanoma (UACC-62)	10.90	1.77	0.22
Breast (MCF-7)	26.41	14.09	2.19
Multidrug resistant ovarian (NCI/ADR-RES)	23.92	14.34	6.19
Kidney (786–0)	32.26	8.45	1.51
Lung (NCI-H460)	26.72	9.55	0.67
Prostate (PC-3)	20.54	5.96	1.15
Ovarian (OVCAR-3)	11.93	3.93	3.78

^a^ Normal cell lines.

**Figure 2:**
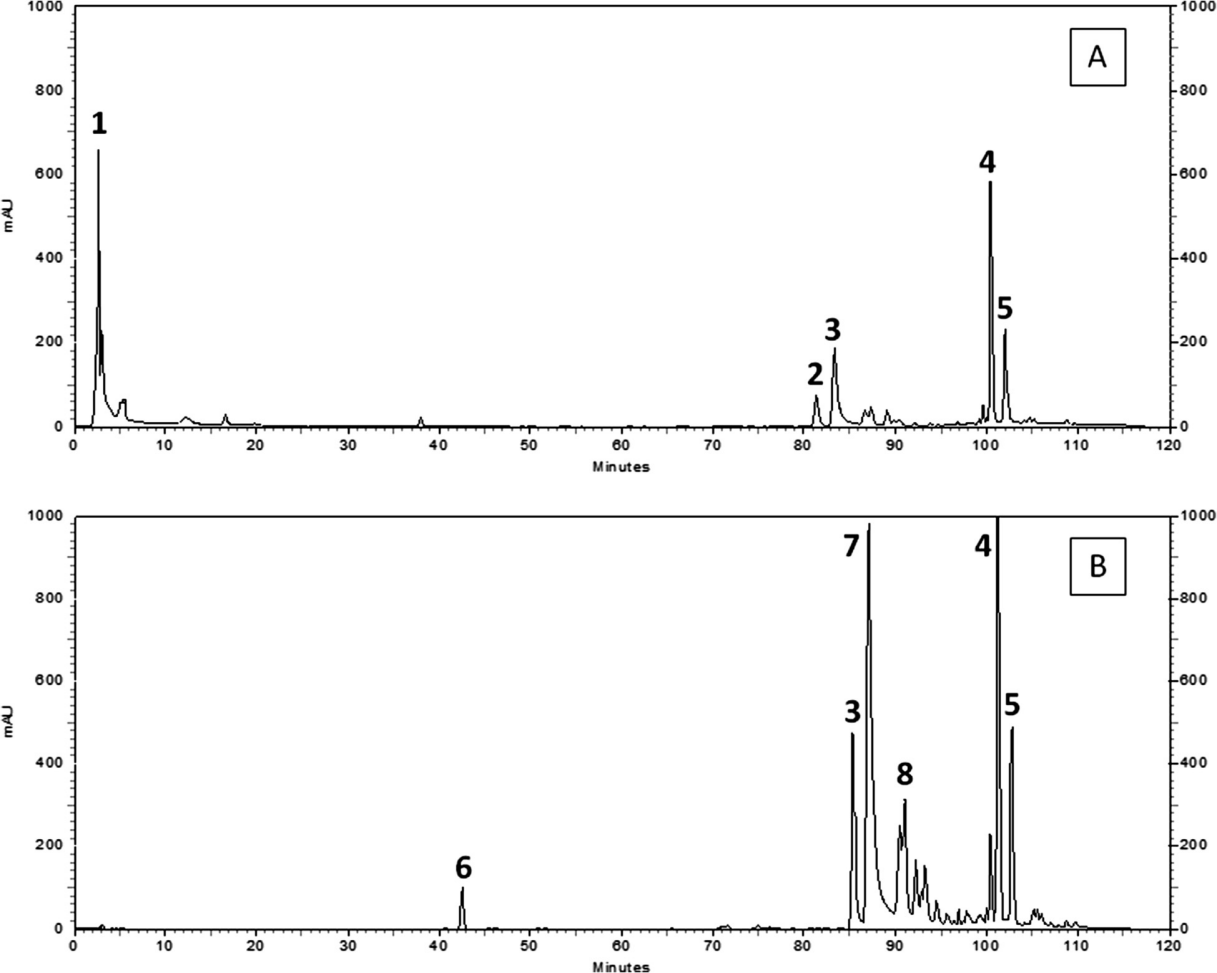
**RP-HPLC chromatograms.** Chromatogram of the ethanolic extract of geopropolis (EEGP) (**A**) and its hexane fraction (HF) (**B**) using photodiode array detector (SPD-M10AVp) at 254 nm; reversed-phase C18 column (250 mm × 4.6 mm i.d.; 5 μm particle size); mobile phase: water/acetic acid (19:1, v/v) (solvent A) and methanol (solvent B) at a constant flow rate of 1 mL/min. The gradient started with 30% solvent B, changing to 40% of B in 15 min, 50% of B in 30 min, 60% of B in 45 min, 75% of B in 65 min, 75% of B in 85 min, 90% of B in 95 min, 90% of B in 110 min, and 30% of B in 120 min. The column was maintained at 35°C. Constituents are represented only by the absorption spectra in the UV (λ_max_): 1: UV λ 241 nm, RT = 2.72 min; 2: UV λ 287 nm, RT = 81.40 min; 3: UV λ 283 nm, RT = 83.31 min; 4: UV λ 279 nm, RT = 100.53 min; 5: UV λ 281 nm, RT = 101.95 min; 6: UV λ 240 nm, RT = 42.54 min; 7: UV λ 282 nm, RT = 87.14 min; 8: UV λ 284 nm, RT = 91.00 min.

The chemical assays were performed for EEGP and HF. The chromatograms obtained by RP-HPLC analyses of EEGP and HF, shown in Figure 2A and B, respectively, demonstrated the presence of similar peaks, however more concentrated in the bioactive fraction (B). No patterns of flavonoid or cinnamic acid derivatives were detected, considering the detection limit of the method. The UV spectrum showed that the major compounds observed, 4, 5, and 7 (Figure 2), have similar λ_max_ at 279, 281, and 282 nm, respectively.

Table 3 shows the compounds identified in EEGP and HF by GC-MS analyses. Most of the substances could not be identified based on the library device, thus confirming the absence of phenolic acid and flavonoid patterns within the detection limit of the method used. Compounds 3 and 4 showed M^+^ at m/z 591, and some fragments at m/z 589, 445, and 73, although with different base peaks (73 and 501, respectively). Moreover, both compounds were more concentrated in the nonpolar fraction (HF), with relative areas of 9.54% and 8.40%. Furthermore, compounds 8 and 9 showed the same M^+^ (m/z 623), similar retention times, and the same fragment m/z 105 ([C_6_H_5_CO]^+^), as well as compound 6. Also, the fragment ions of m/z 77, 69, and 55 were observed in compound 6, while compounds 8 and 9 showed fragments m/z 69 and 77, respectively. Compound 8 was the most abundant one found in EEGP and HF, and compound 9 was more concentrated in EEGP compared to HF. According to the results of HPLC, no flavonoids were found.

**Table 3:** Kovats retention index (RI), retention times (RT), concentration of each component (relative area), and important ions present in the mass spectra of compounds of the identified compounds in the ethanolic extract of geopropolis (EEGP) and its hexane fraction (HF) by gas chromatography–mass spectrometry (GC-MS)

**Compound**	**RI**	**RT (min)**	**Relative area (%)**		**Major MS peaks: m/z**
			**EEGP**	**HF**	
2-propensaeure 3-phenyl-trimethylsilylester	1542	17.84	8.91	3.92	220, 205, 161, 145, 131, 103, 77
1,2-benzenedicarboxylic acid	2543	36.60	ND^a^	2.24	167, 149, 57
3	–	43.06	0.68	9.64	591, 589, 499, 445, 73
4	–	43.79	6.88	8.40	591, 589, 501, 459, 445, 73, 57
5	–	46.38	3.34	9.06	533, 386, 177, 165, 151, 138, 105, 77, 69, 55
6	–	46.94	5.79	2.30	495, 459, 417, 105, 77, 73, 69, 57, 55
7	–	47.29	19.17	10.35	548, 533, 479, 389, 73, 45
8	–	47.71	29.15	38.98	623, 536, 535, 105, 73, 69
9	–	47.91	6.93	4.76	623, 533, 551, 461, 407, 105, 77, 73

^a^ Non detected compound.

## Discussion

Propolis, a resin collected by bees, exhibits a considerable variety of well-established pharmacological activities, and its antimicrobial potential has been widely studied, especially against oral pathogens [1,14,15,19]. Most of these studies describe the activity of propolis collected by *A. mellifera*, which increased the market price of this product. Geopropolis is a type of propolis collected by stingless bee species native to tropical countries, which, in addition to resin and wax, has soil in its composition, leading to low yield extracts, a fact that can partly justify its low economic value and the lack of studies on its biological activity [3].

In this study, EEGP showed interesting antimicrobial activity, especially against *S. aureus*, *S. mutans*, and MRSA strains, with MIC values below 50 μg/mL, but it presented weak inhibition of *P. aeruginosa* growth, a Gram-negative bacillus. Our findings are corroborated by Velikova et al. [4], who reported that Brazilian geopropolis samples showed significant activity against *S. aureus* but presented weak activity against *E*. *coli*, as well as by Duarte et al. [20], who affirmed that crude extracts from natural products are considered promising when the MIC value is below 500 μg/mL.

Several types of *A. mellifera* propolis extracts have their activity against *S. mutans* well described in the literature. Duarte et al. [21] showed that the ethanolic extract of Brazilian propolis type 6 inhibited *S. mutans* UA 159 growth at concentrations between 25 and 100 μg/mL and Hayacibara et al. [19] reported that Brazilian propolis types 3 and 12 were able to inhibit bacterial growth at 25–50 μg/mL and 200–400 μg/mL, respectively. EEGP inhibited the growth of *S. mutans* UA 159 between 25 and 50 μg/mL, also demonstrating strong inhibitory activity with bacteriostatic character, suggesting its ability to act on the virulence factors of the microorganisms involved in the etiology of dental caries. In case of an infection in the oral cavity, actions that have impact on the virulence factor of the microorganisms seem to be the best way to control their development and pathogenesis, since total and permanent elimination of bacteria from the oral environment is not viable [22]. Such effect of geopropolis, provided that it is confirmed by specific studies, indicates the presence of compounds that can be effective in controlling and preventing caries. *S. aureus* and MRSA infections have acquired great clinical importance, because these organisms appear to be resistant to β-lactam, aminoglycoside, and macrolide antibiotics as well as to certain antiseptic substances [23]. In the present study, EEGP demonstrated to be a promising source of bioactive compounds against this pathogen, showing the lowest MIC and MBC values against both *S. aureus* strains tested. Furthermore, when compared to other strains, MRSA was the most sensitive microorganism, with all fractions tested showing low MIC and MBC values.

In order to verify whether the chemical separation was efficient, HF, CF, and EAF were tested against the same microorganisms and their MIC values were compared to the values obtained for EEGP. HF proved to be the most potent fraction, reducing MIC and MBC values (between two to four times) for *S. mutans*, *E. faecalis*, and *A. naeslundii,* and maintaining these values against *S. aureus* 25923 compared to EEGP. Against MRSA, HF was less active than EEGP and CF. In general, all the other fractions showed low bacterial growth inhibition compared to HF and EEGP. Such effect suggests that nonpolar compounds present in geopropolis should be the main substances responsible for its biological activity.

EEGP and HF (defined as the most active fraction) were also able to inhibit the adherence of *S*. *mutans* growing cells at sub-MIC concentrations. The inhibition rates observed indicate that HF probably presents higher 2activity than EEGP because of a concentration of bioactive compounds in the nonpolar fraction, which suggests that this fraction might contain promising anti-caries agents. Other types of Brazilian propolis also showed this antibiofilm activity with a similar mechanism of action, especially Brazilian propolis type 6, which presented activity against adherence of *S*. *mutans* growing cells due to its activity on glucan synthesis by inhibiting glucosyltransferases [14]. Furthermore, other kinds of geopropolis collected by other bee species and from different regions demonstrated similar mechanisms of action against *S*. *mutans*, reducing the cell viability of the biofilm formed by this microorganism [5].

According to Fouche et al. [24], extracts of natural products with antiproliferative activity can be classified as inactive (TGI > 50 μg/mL), presenting weak activity (15 μg/mL < TGI < 50 μg/mL), moderate activity (6.25 μg/mL < TGI < 15 μg/mL), and potent activity (TGI < 6.25 μg/mL). EEGP was inactive against normal murine fibroblast cells and a weak inhibitor of human keratinocytes. Among the human cancer cell lines tested, EEGP showed moderate inhibition against melanoma and ovarian cancer lines. These results indicate that EEGP exhibited a nontoxic profile against normal cell lines and toxicity against cancer cell lines, i.e., a selective antiproliferative activity. Additionally, HF maintained the weak activity against HaCaT cells, promoted a six-fold reduction in TGI value against the melanoma line compared to EEGP, and presented potent activity against prostate and ovarian tumors.

Umthong et al. [10] described the selective antiproliferative activity of propolis collected by *Trigona laeviceps*, a stingless bee species, against some cancer cell lines and low cytotoxic activity against normal cell lines. Comparatively, *M. scutellaris* geopropolis seems to be a promising source of anti-tumor bioactive compounds, showing moderate or strong inhibition of a wide range of cell lines. Although these are preliminary results obtained from *in vitro* evaluations, they indicate that the compounds present in EEGP and HF could be used to treat some types of infections and tumors without causing significant damage to the normal cells tested here. In fact, the concentrations of EEGP and HF that can affect the normal cell lines were higher than those effective against some bacteria or tumor cell lines.

RP-HPLC analyses confirmed the presence of low polarity compounds in *M. scutellaris* geopropolis, evidenced by high elution times (RT between 80 and 120 min), corresponding to less polar compounds, and also by the concentration of substances in HF. Other types of propolis and Brazilian geopropolis are essentially nonpolar due to the presence of terpenes and benzophenones [4]. The UV spectra of the major compounds (4, 5, and 7, Figure 2) ranged from λ_max_ 279 to 282 nm, suggesting a possible chromophore with characteristics of polyprenylated benzophenones [25]. Our findings also indicate the absence of flavonoids, usually reported as responsible for the pharmacological activities attributed to some types of *A. mellifera* propolis, as well as of markers of some types of propolis [3,26].

CG-MS data showed the presence of compounds belonging to similar chemical classes, indicated by the fragmentation pattern of the mass spectra. The fragment at m/z 105 [(C_6_H_5_CO) ^+^, observed in the fragmentation pattern of compounds 6, 8, and 9, suggests that they have characteristics of the class of benzophenones, and the presence of fragments at m/z 55, 69, and 77 indicates that they present prenylations [27]. These findings about the chemical profile of *M. scutellaris* geopropolis corroborate the differentiated profile and the not yet entirely elucidated nature of its bioactive compounds. This stimulates the search for a detailed description of its chemical composition and the potential use of its bioactive compounds as complementary food or medicine, thus increasing the economic and social value of a natural product not fully recognized.

The presence of benzophenones, especially polyprenylated ones, has been described in some types of propolis. Ishida et al. [25] attributed the antimicrobial activity of propolis samples from the Brazilian Amazon region to benzophenones, such as *epi*-nemorosone and 7-*epi*-clusianone, which are also described as typical metabolites produced by Clusiaceae (Guttifarae), a family of plants widely distributed in Brazil. Studies on the chemical composition and biological activity of Brazilian propolis type 6, collected by *A. mellifera* in the state of Bahia, showed certain similarities to the geopropolis studied herein, from the same state, although they were collected by bees with completely different biological characteristics [21,28]. The studies reported that Brazilian propolis type 6 has an essentially nonpolar composition, showing the possible presence of benzophenones and the absence of phenolic acids and flavonoids [21,28]. Similarly to the geopropolis of the present study, HF was the fraction responsible for the best activity of Brazilian propolis type 6, and its biological activity was attributed to hyperibone A, which also acts against the adherence of *S. mutans* biofilm [15]. The similarities between the chemical and biological profiles of geopropolis collected by *M. scutellaris* and Brazilian propolis type 6 suggest that the possible activity of the former is due to the presence of a benzophenone.

## Conclusion

Geopropolis collected by *M. scutellaris* presented interesting antimicrobial and antiproliferative activity. Also, it proved to be a promising source of antibiofilm agents and to present selectivity against human cancer cell lines at low concentrations compared to normal cells. Its chemical composition appears to be essentially nonpolar, which is confirmed by the concentration of its activity in low polarity fractions. Moreover, the characteristics evidenced by the chemical analyses suggest the presence of benzophenones as active compounds. Therefore geopropolis seems to be a promising natural product to be thoroughly studied in order to reveal new molecules with therapeutic properties, since its chemical profile has not been fully described and its pharmacological potential has just begun to be unveiled and deserves further studies.

## Competing interests

The authors have no competing of interests.

## Authors’ contributions

MGC, MF, and LCCG participated in the design of the study, carried out the extraction and fractionation of geopropolis, the antimicrobial tests, and performed the statistical analysis. ALTGR and JEC carried out and interpreted the antiproliferative study. MI and SMA carried out and interpreted the chemical analyses. HK and PLR participated in the design of the study and prepared the article for publication. All authors read and approved the final manuscript.

## Pre-publication history

The pre-publication history for this paper can be accessed here:

http://www.biomedcentral.com/1472-6882/13/23/prepub
